# Atypical 15q11.2-q13 Deletions and the Prader-Willi Phenotype

**DOI:** 10.3390/jcm11154636

**Published:** 2022-08-08

**Authors:** Lionne N. Grootjen, Alicia F. Juriaans, Gerthe F. Kerkhof, Anita C. S. Hokken-Koelega

**Affiliations:** 1 Dutch Reference Center for Prader-Willi Syndrome, 3015 CN Rotterdam, The Netherlands; 2 Department of Pediatrics, Subdivision of Endocrinology, Erasmus University Medical Center-Sophia Children’s Hospital, 3015 CN Rotterdam, The Netherlands; 3 Dutch Growth Research Foundation, 3016 AH Rotterdam, The Netherlands

**Keywords:** Prader-Willi syndrome, genotype–phenotype correlation, atypical deletions, SNURF-SNPRN, SNORD116

## Abstract

Background: Prader-Willi syndrome (PWS) is a rare genetic disorder resulting from the lack of expression of the PWS region (locus q11-q13) on the paternally derived chromosome 15, as a result of a type I or II paternal deletion (50%), maternal uniparental disomy (43%), imprinting defect (4%) or translocation (<1%). In very rare cases, atypical deletions, smaller or larger than the typical deletion, are identified. These patients may have distinct phenotypical features and provide further information regarding the genotype–phenotype correlation in PWS. Methods: A prospective study in eight patients (six males and two females) with an atypical deletion in the PWS region accompanies an overview of reported cases. Results: All patients had hypotonia (100%) and many had typical PWS facial characteristics (75%), social and emotional developmental delays (75%), intellectual disabilities (50%), neonatal feeding problems and tube feeding (63%), history of obesity (50%), hyperphagia (50%) and scoliosis (50%). All males had cryptorchidism. Two patients had two separate deletions in the PWS critical region. Conclusions: Our findings provide further insight into PWS genotype–phenotype correlations; our results imply that inclusion of both SNURF-SNPRN and SNORD-116 genes in the deletion leads to a more complete PWS phenotype. A larger deletion, extending further upstream and downstream from these genes, does not cause a more severe phenotype. Conventional PWS methylation testing may miss small deletions, which can be identified using targeted next generation sequencing. PWS’s phenotypic diversity might be caused by differentially methylated regions outside the 15q11.2 locus.

## 1. Introduction

Prader-Willi syndrome is a rare genetic disorder with an estimated incidence of 1:10,000–1:30,000 live births [1]. The syndrome is characterized by hypotonia leading to poor feeding and failure to thrive in neonates, followed by hyperphagia in childhood, which leads to obesity when intake is unrestricted [2,3]. In addition, an abnormal body composition, with a higher fat mass and a lower lean body mass, is one of the core symptoms [4]. PWS also includes endocrine problems, such as hypogonadism and growth hormone deficiency. Most of these symptoms are caused by hypothalamic dysfunction. Other symptoms of PWS include delayed development, cognitive impairment and behavioral problems [4]. In addition, patients with PWS often present with typical facial features, such as almond-shaped eyes, strabismus, narrow bitemporal diameter, thin upper lip with a tent-shaped mouth and small hands and feet [1,3]. However, the PWS phenotype is extremely varied among patients.

PWS results from the lack of expression of the PWS region (locus q11-q13) on the paternally derived chromosome 15 [1,2,4] due to a paternal deletion of the PWS region (50–75%), a maternal uniparental disomy (mUPD; 20–44%), an imprinting defect (<5%) or translocation (<1%) [5,6,7,8]. Most cases (>99%) can be easily detected using DNA methylation analysis to detect deletions or abnormal methylation of the PWS region [9]. The PWS region includes several genes, such as MKRN3, MAGEL2, NDN, NPAP1 and SNURF-SNRPN, as well as non-coding RNAs, including small nucleolar RNAs (snoRNAs) [10]. However, the relationship between the lack of expression of these genes and variations in the PWS phenotype remains unclear.

Deletions are usually de novo and manifest either as a large type I deletion or a smaller type II deletion. The larger type I deletion involves breakpoint (BP) 1 and BP3 (±6 Mb); the type II deletion covers two distal BPs (BP2 and BP3) (±5.6 Mb). In more rare cases, atypical deletions, smaller or larger than the typical type I and II deletions, are identified. Cases with atypical deletions can provide insight into PWS genotype–phenotype correlations. Several case reports suggest that disruptions in the SnoRNA cluster SNORD116 cause PWS’s primary characteristics [11,12,13,14,15,16,17]. However, a PWS phenotype has been reported in patients with only a SNURF-SNRPN aberration consisting of either a small deletion [18,19] or a point mutation [20,21].

We report eight patients with an atypical deletion found in our Dutch PWS cohort. Together with published reports of patients with atypical deletions, these cases provide further information towards unravelling the mystery of the genotype–phenotype correlations of PWS.

## 2. Materials and Methods

### 2.1. Patients

Patients who visited the Dutch Reference Center for Prader-Willi syndrome were eligible to be included in the present study. We included patients who were diagnosed with an atypical deletion in the PWS region.

### 2.2. Design and Data Collection

Data regarding patients’ neonatal histories and genetic diagnoses were available from medical records. Physical features were observed during regular examinations by the PWS team. Standing height was measured using a Harpenden Stadiometer and weight was measured using a calibrated electric scale (Servo Balance KA-20-150S; Servo Berkel Prior, Katwijk, The Netherlands). Height, weight and BMI standard deviation scores (SDS) were calculated using Growth Analyser RCT based on Dutch reference values [22,23]. IQ scores were assessed using the Wechsler Adult Intelligence Scale III-NL (WAIS-III), the Wechsler Intelligence Scale for Children V (WISC-V-NL) or the Wechsler Preschool and Primary Scale of Intelligence III (WPPSI-III-NL), depending on the age of the patient. Hyperphagia and behavioral problems of the eight patients were scored based on a structured interview with parents carried out by two physicians (A.F.J and L.N.G). Written informed consent was obtained from parents and from patients aged 12 years and older. Assent was obtained from patients younger than 12 years. Data are presented as median and interquartile range (IQR), which were calculated using SPSS Inc, Chicago, IL, USA, version 25.0.

### 2.3. Genetic Testing

Cytogenetic analysis was performed on genomic DNA obtained from patients’ peripheral blood. DNA was hybridized using an Illumina Infinium GSA+MD-24 v3.0 BeadChip and analyzed for the presence of copy number variants.

The ME028-C1 PWS/AS probemix kit by MRC-Holland, was used for the methylation specific multiplex ligation-dependent probe amplification assay (MS-MLPA) (http://www.mrc.holland.com).

### 2.4. Literature Review

Literature libraries were searched for relevant publications dated from January 1963 to January 2022. Relevant publications included case reports and case series of atypical deletions in the PWS region of the paternal chromosome, caused by either a deletion or a translocation. All case reports and series with a detailed description of the genotype and phenotype were included. Papers that did not explicitly describe the location of the deletion or the case’s clinical information were excluded. Papers describing cases with balanced translocations were also excluded. In total, 20 papers and 29 cases were included in our literature overview [5,11,12,13,14,15,16,17,18,19,20,21,24,25,26,27,28,29,30,31,32].

## 3. Clinical Reports

**Figure 1:**
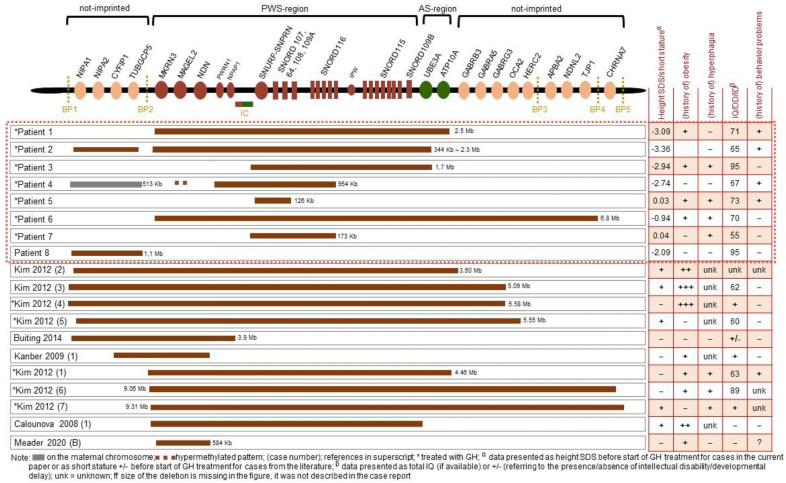

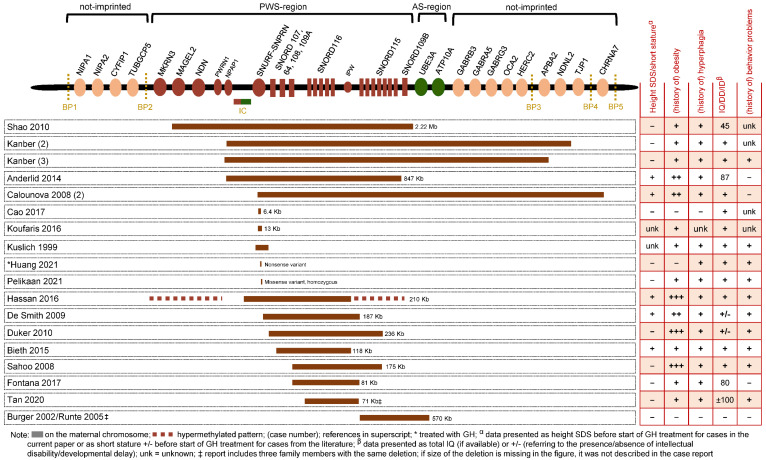
Size of the deletion and location per patient [5,11,12,13,14,15,16,17,18,19,20,21,24,25,26,27,28,29,30,31,32].

Patients’ clinical reports are summarized in Table 1. Sizes and locations of deletions for patients 1 to 8 are depicted in Figure 1, which also provides an overview of all eligible cases reported in the literature, including the sizes and locations of deletions in every case.

### 3.1. Patient 1

#### 3.1.1. Clinical Characteristics

Patient 1, a boy, was born after 42 weeks of gestation with a birth weight of 2800 g (−2.3 SDS). He had hypotonia, feeding difficulties, cryptorchidism and received tube feeding. Weight gain started at age 3 years, leading to obesity. His weight normalized with a strict diet intervention. PWS was diagnosed at age 4 years. Growth hormone (GH) treatment started at age 14.9 years. He had scoliosis, which was treated with brace therapy until he reached adult height. Hypogonadotropic hypogonadism was treated with testosterone therapy. In adolescence, he developed anxiety, a mood disorder and aggressive behavior, for which he received risperidone treatment.

At his most recent visit, at age 30.3 years, his height was 175.0 cm (−1.3 SDS), his weight was 61.3 kg (−0.4 SDS) and his BMI was 20.0 kg/m^2^ (−0.9 SDS). He had a narrow bifrontal diameter, thin upper lip and flat, long philtrum. Hyperphagia was no longer present. Cognitive testing using the WAIS-III revealed a full scale IQ of 87.

#### 3.1.2. Genetic Testing

Genetic testing was repeated at age 28 years using MS-MLPA analysis, which showed a deletion with the same breakpoint as a type II deletion on the proximal end and a distal breakpoint between ATP10A and GABRB3, which is upstream to BP3 and thus atypical. SNP array results confirmed the ~4.5 mb deletion, spanning the chr15: 23616115-26146992 region in the GRCh37/hg19 build of the human genome. The parents’ chromosome patterns were not further analyzed. 

### 3.2. Patient 2

#### 3.2.1. Clinical Characteristics

Patient 2, a boy, was born in the breech position after 41 weeks gestation with a birthweight of 2430 g (−3.2 SDS). He had cryptorchidism, hypotonia and required tube feeding. PWS was diagnosed at age 6 months. At age 2 years, he had strabismus and was diagnosed with hypothyroidism, upon which treatment with levothyroxine started. GH treatment started at age 7.9 years. He had a tendency to become overweight, but weight gain control was successful. He had no hyperphagia, but did follow a strict diet protocol. He started exhibiting challenging behavior, including obsessive-compulsive behavior, aggression and temper tantrums, at age 12 years. He moved to a group home at age 16, and began risperidone treatment. He had a severe kyphosis with a Cobb angle of 70° and hypogonadotropic hypogonadism, for which he received testosterone replacement therapy.

At his last visit, at age 24.7 years, his height was 172.8 cm (−1.6 SDS), his weight was 65.2 kg (0.3 SDS) and his BMI was −0.1 SDS. He had several distinctive facial features, including a narrow bifrontal diameter, strabismus and low implanted ears. Cognitive testing using the WAIS-III revealed a full scale IQ of 65.

#### 3.2.2. Genetic Testing

Genetic testing was performed using MS-MLPA analysis, which revealed a deletion in the PWS region. The proximal breakpoint was mapped to BP1 and the distal breakpoint was mapped to the UBE3A gene. Probes for ATP10A, GABRB3 and OCA2 showed a normal pattern. SNP array results revealed that the initial deletion actually consisted of two losses. The first loss, mapped from BP1 to BP2, was 344 Kb in length and located at genomic coordinates chr15:22761722-23105656 (hg19). The second loss was 2.3 Mb; its proximal breakpoint was at the MKRN3 gene and its distal breakpoint was at the UBE3A gene located at genomic coordinates chr15:23616115-25876991. The parents’ chromosome patterns were not further analyzed.

### 3.3. Patient 3

#### 3.3.1. Clinical Characteristics

Patient 3, a boy, was born after 42 weeks of gestation with a birth weight of 3260 g (−1.2 SDS). He suffered from hypotonia and feeding difficulties, but did not require tube feeding. He had cryptorchidism. PWS was diagnosed at approximately age 1 year. His motor milestones were slightly delayed. GH treatment started at age 3.6 years. He attended mainstream primary and secondary schools and started vocational training thereafter. At age 17 years, his body weight started to increase and he had hyperphagia. He received testosterone treatment due to hypogonadotropic hypogonadism.

At his last visit, at age 22 years, his height was 181.5 cm (−0.4 SDS), his weight was 124.9 kg (+3.4 SDS) and his BMI was 3.7 SDS. He did not show any facial characteristics of PWS. Cognitive testing using the WAIS-III revealed a full scale IQ of 95.

#### 3.3.2. Genetic Testing

Prader-Willi syndrome was diagnosed using MS-MLPA analysis and repeated at age 18 years. MS-MLPA results showed a deletion spanning SNRPN to UBE3A. SNP array results revealed a deletion of 1.7 Mb, located at genomic coordinates (hg19) chr15: 24106835-25843032. Karyotyping the parents’ DNA revealed a normal pattern in the mother and a paracentric inversion of chromosome 13 in the father (who had no clinical features), which was also found in Patient 3.

### 3.4. Patient 4

#### 3.4.1. Clinical Characteristics

Patient 4, a girl, was born after 40 weeks of gestation with a birth weight of 2430 g (−2.7 SDS). Prenatal ultrasound indicated intra-uterine growth retardation (IUGR) and a thickened heart muscle with a mild tricuspid regurgitation. She presented with hypotonia and required tube feeding after birth. Postnatal follow-up did not reveal any underlying cardiac diseases. PWS was diagnosed before the age of 1 month. GH treatment was started at age 8 months. Her motor milestones were delayed. At age 5.8 years, she developed a scoliosis, which was treated surgically and required two additional surgeries at a later age.

At age 6 years, her behavioral problems began to increase and risperidone treatment was started. At approximately age 6 years, she began to gain weight and became overweight, but she was never obese. She did not have hyperphagia. At age 9 years, her behavioral problems became unmanageable. She moved to a group home for people with PWS, where she followed a strict daily routine, which had a positive impact on her behavior and wellbeing. Her weight also dropped to a healthy range.

At her last visit, at age 12.1 years, her height was 142.6 cm (−2.0 SDS), her weight was 42.1 kg (1.8 SDS) and her BMI was 1.0 SDS. Facial characteristics included downturned corners of the mouth, upslant of the eyes and a thin upper lip. Cognitive testing using the WPPSI-III-NL revealed a full scale IQ of 67.

#### 3.4.2. Genetic Testing

Genetic testing via MS-MLPA showed two deletions in chromosome 15. The distal deletion included all probes for the SNRPN gene and SNORD116. However, probes for MKRN3, MAGEL2, NDN, UBE3A, ATP10a, GABRB3, OCA2 and APBA2 showed a normal pattern. Methylation of the probes for SNPRN (included in the deletion) and MAGEL2 (not included in the deletion) showed a pattern of hypermethylation. In addition, a proximal deletion, at minimum including genes TUBGCP5 and NIPA1, was found.

Further genetic analysis using an SNP array revealed a gain of 591 kb in 11q25 (chr15:22766739-23279684 in hg19), a proximal loss of 513kb in 15q11.2 (chr15: 22766739-23279684 in hg19) and a distal loss of 954 kb in 15q11.2 (chr15: 24369482-25323964 in hg19). The 11q25 gain was also found in her father, who has no clinical features of PWS. The proximal deletion from BP1 to BP2 is known to cause a variable phenotype [33,34] and was also found in Patient 4’s mother, who was asymptomatic. Fluorescence in situ hybridization was performed in her father to exclude the presence of a balanced insertion, which could be the underlying cause of the proximal 15q11.2 deletion. No insertion was detected. No other genetic testing was performed. The distal deletion in the PWS region was de novo and found on the paternally derived chromosome.

### 3.5. Patient 5

#### 3.5.1. Clinical Characteristics

Patient 5, a girl, was born after an uncomplicated pregnancy of 41 weeks of gestation with a birthweight of 2765 g (−1.9 SDS). She was born by cesarean section, due to non-progressive labor and fetal distress. After birth, muscular hypotonia was present, but she did not require tube feeding. She was diagnosed with hip dysplasia. PWS was diagnosed at age 1.8 years. GH treatment started at age 2.5 years, which is also when weight increase began. At the age of 5 years, she was obese. She attended special education and had mild behavioral problems. She was described as being insatiable, with an extreme focus on food.

At her last examination, at age 11.9 years, her height was 160.0 cm (0.8 SDS), her weight was 72.1 kg (1.8 SDS) and her BMI was 2.5 SDS. Cognitive testing using WISC-V-NL revealed a full scale IQ of 73.

#### 3.5.2. Genetic Testing

Genetic testing using MS-MLPA showed abnormal patterns for several of the SNPRN exons. At age 12 years, SNP array results revealed a minor deletion of 126 kb, encompassing only SNRPN and a few non-coding genes (chr15: 25153293-25279455 in hg19). Her father’s DNA was analyzed to determine if he carried the 15q11.2 microdeletion; he did not.

### 3.6. Patient 6

#### 3.6.1. Clinical Characteristics

Patient 6, a boy, was born after 39 weeks of gestation with a birth weight of 2525 g (−1.7 SDS). After birth, he suffered from hypotonia, tachypnea, feeding difficulties and cryptorchidism. PWS was diagnosed at age 3 weeks. GH treatment started at age 0.6 years. Motor milestones were delayed. At age 2 years, he was diagnosed with scoliosis, which required surgical intervention. He received special education. Hyperphagia and weight increase started at approximately age 5 years.

At his last visit, at age 9.1 years, his height was 137.6 cm (−0.2 SDS), his weight was 51.8 kg (3.5 SDS) and he had a BMI of 3.0 SDS. Cognitive testing using WPSSI-III-NL revealed a full scale IQ of 70.

#### 3.6.2. Genetic Testing

Genetic analysis using MS-MLPA showed a type 2 deletion in the PWS region, which was longer on the telomeric side of the chromosome; the probe for APBA2 also showed a hypermethylated pattern. SNP array results confirmed the abnormal methylation pattern of the APBA2 gene; the distal breakpoint of the deletion was mapped to BP4. The deletion was 6.8 Mb in size and located at genomic coordinates 23616115–30369914 on chromosome 15 (hg19). The parents’ chromosome patterns were not further analyzed.

### 3.7. Patient 7

#### 3.7.1. Clinical Characteristics

Patient 7, a boy, was born after 33 weeks of gestation with a birth weight of 2195 g (0.3 SDS). He needed assisted ventilation immediately after birth, had muscular hypotonia and cryptorchidism. He had a narrow, triangular face, a small mouth with thin lips and mandibular retrognathia. He required tube feeding until age 5 months. PWS was diagnosed at approximately age 3 weeks; he started GH treatment at age 7 months.

At his most recent examination, at age 4.2 years, his height was 115 cm (1.9 SDS), his weight was 24.5 kg (1.8 SDS) and his BMI was 1.9 SDS. He had typical PWS facial features. He had food-seeking behavior and an increased focus on food. He was non-verbal, but able to convey his needs. He went to a specialized daycare for children with developmental delays. Cognitive testing using WPSII-III-NL revealed a full scale IQ < 55. An X-ray of the spine revealed a minimal scoliosis of 16 degrees.

#### 3.7.2. Genetic Testing

SNP array results showed a male pattern with a 173 kb loss within 15q11.2. A methylation specific PCR confirmed that the paternal allele was deleted; further investigation of the deletion revealed a loss of SNURF-SNPRN exon 3, extending to the SNORD116 snoRNA cluster. The deletion was located at genomic coordinates (hg19) chr15:25192442-25365030. Targeted arrays and trio analyses were performed on his parents, which revealed no abnormalities.

### 3.8. Patient 8

#### 3.8.1. Clinical characteristics

Patient 8, a boy, was born after 38 weeks gestation; his mother’s pregnancy was complicated by diabetes gravidarum and hypertension. His birth weight was 3266 g (0.14 SDS) and his birth length was 48 cm (−0.69 SDS). He did not require tube feeding after birth, but had notable muscular hypotonia. During infancy it became apparent he had a developmental delay. Motor milestones were reached late and his speech remained unintelligible.

At his last examination, at age 4 years, his height was 97.1 cm (−2.1 SDS) and his weight was 14.1 kg (−0.8 SDS). He had not yet started GH treatment. He had mild dysmorphic features, including a prominent forehead, triangular face and simian crease on his left hand. His motor development had improved, but his speech was still delayed. He was easily startled and overstimulated, but there were no behavioral problems. The WPPSI-III-NL test revealed a full scale IQ of 95.

#### 3.8.2. Genetic Testing

Patient 8 was evaluated by a pediatrician at age 2 years due to psychomotor delay; SNP array results revealed a 1.1 mb deletion in 15q11.2 from BP1 to BP2 at genomic coordinates (hg19) chr15:22320346-23447898 and a gain in 6q27 of 561.5 kb at genomic coordinates chr6:167619453-168180979. The parents’ chromosome patterns were not further analyzed.

## 4. Discussion

PWS is caused by the lack of expression of the paternally inherited genes in the 15q11.2 region. Typical defects include type 1 and type 2 deletions which span from BP1 to BP3 and BP2 to BP3, respectively. Studying patients with deletions other than the typical type 1 and 2 might provide insight into the role of individual PWS-region genes in the phenotype. In this study, we aimed to find a link between the genotype and the phenotype; however, our elaborate data, including our overview of cases described in the literature, did not reveal an evident correlation. This could imply that an explanation for the variation in phenotype might lie outside the 15q11.2 region.

Our study details eight patients with atypical deletions in the PWS region. All patients exhibited a PWS phenotype, with notable differences. All patients presented with hypotonia (100%) and male patients presented with cryptorchidism (100% of boys). Many patients presented with typical PWS facial characteristics (75%), social and emotional developmental delay (75%), intellectual disability (50%), neonatal feeding problems and tube-feeding (63%), (history of) obesity (50%), hyperphagia (50%) and scoliosis (50%).

Several studies have attempted to narrow down the minimal critical region for PWS, suggesting disruption of the paternally inherited SNURF-SNPRN as being sufficient to cause the PWS phenotype [18,19,35,36,37]. In this study, seven of eight patients had a deletion that included SNURF/SNPRN. Patient 8’s deletion did not include SNURF-SNPRN; his deletion was upstream to SNURF-SNPRN, from BP1 to BP2, and 1.1 Mb in length. His symptoms included failure to thrive and short stature (his target height was within the normal range). He had an IQ of 95 and lacked a few distinct PWS symptoms, such as tube feeding, but did have hypotonia. He had neither obesity nor hyperphagia, but was still young. Although obesity and hyperphagia may appear at a later age in Patient 8, PWS-related weight gain typically occurs around age 4 years [38]. The deletion in Patient 5 did include SNURF-SNRPN, but it was very small (126 kb), encompassing only a few exons of SNPRN and a small number of non-coding genes (SNORD107-SNHG14); the deletion did not include SNORD115 or SNORD116. Patient 5 had an average height and lacked certain distinctive PWS features, such as severe hypotonia in infancy and typical PWS facial features; however, she did have obesity and hyperphagia. She had a below average IQ of 73 and was enrolled in special education. The patient presented by Cao et al. was also diagnosed with an exceptionally small (6.4 kb) deletion of SNURF/SNPRN exon 1, the smallest deletion in the region to be reported to date. She had several major PWS symptoms, such as hypotonia, dysmorphic features, and intellectual disability, and was overweight, but had an average height [18]. Two other case reports presented two patients with a small deletion in SNURF-SNPRN and a PWS phenotype including hypotonia, intellectual disability and obesity, but did not mention short stature. In two recent reports describing patients with a PWS phenotype and point mutations in SNURF-SNPRN [20,21], both patients presented with a typical PWS phenotype, including hypotonia and feeding difficulties in infancy, intellectual disability, hyperphagia and a tendency to become overweight in later childhood. However, one of the patients had an average height and the other had a low-normal height. This is similar to our Patient 5 and the patient presented by Cao et al. Thus, our cases and several case reports imply that intellectual disability in PWS might be linked to disruption of the SNURF-SNPRN gene. However, patients with larger deletions in the PWS region can also have an average IQ, as shown by our Patient 3. None of the patients with a small deletion or point mutation in SNURF-SNPRN had short stature, which could mean that this feature of PWS might not be caused by disruption or deletion of the SNURF-SNPRN gene.

Recent case reports suggest that deletions in the snoRNA cluster, specifically in the SNORD116 gene, are the main causes of the PWS phenotype [11,12,13,14,15,17]. None of our patients had a loss limited to the snoRNA cluster, but it was included in many patients’ deletions. Patient 8’s deletion did not include any of the snoRNA genes. He was noticeably different from the other patients, as he had an IQ of 95 and did not have hyperphagia or obesity. However, he did have notable hypotonia, mild PWS facial features and short stature. In contrast, Patient 5, whose deletion only included SNURF-SNPRN and a few of the most proximal snoRNAs (without SNORD116), was not very different from the other patients and had an IQ of 73. In six of eight patients, the SNORD116 genes were included in the deletion. Patients 4 and 7 had a deletion that included SNURF-SNPRN and a larger part of snoRNA cluster, including the SNORD116 genes. Patient 7 had a relatively small deletion of 173 Kb, spanning from SNURF-SNPRN to SNORD116. He was considered low functioning with a relatively low IQ and evident hyperphagia. Patient 4 had a more complicated genotype, with two deletions in the PWS region. The second deletion included SNURF-SNPRN and SNORD116, as well as the imprinting center; the first deletion included the non-imprinted genes NIPA1, NIPA2, CYFIP1 and TUBGCP5. She was low functioning and had severe behavioral problems, intellectual disability and severe scoliosis. Patient 4’s hyperphagia might have been clinically masked because she followed very strict rules and had severe disabilities; as a result, she was unable to seek food, which prevented her from developing obesity. Patients 1, 2, 3 and 6 had relatively large deletions encompassing the entire snoRNA cluster and, in some cases, more genes downstream to the snoRNA cluster. However, their phenotypes appeared to be no more severe than that of patients 4, 5 and 7, who had smaller deletions including SNURF-SNPRN and SNORD116. Therefore, larger deletions, extending beyond SNURF-SNPRN and SNORD116, do not seem to cause a more severe phenotype; inclusion of the SNORD115 cluster does not appear to be essential for a PWS phenotype.

It has been proposed that deletions limited to SNORD116 might cause a milder, atypical PWS phenotype [17]; previous studies confirm that deletions restricted to the SNORD115 genes do not cause symptoms [31,32]. Many patients with atypical deletions limited to the SNORD116 genes have normal stature [12,13,17] and average intellectual development [13,17]. There is some evidence that the SNORD116 cluster is highly expressed in the hypothalamus; studies in mice have shown that it plays a role in regulating appetite [39]. The prevalence of small deletions in the snoRNA cluster might be underestimated, because they can be missed by the classical PWS DNA methylation analysis; their discovery would require targeted testing or WES (whole exome sequencing) analysis.

Two of our patients (2 and 4) had two deletions in the PWS region. Both patients had a deletion in the non-imprinted genes and a second deletion in the imprinted genes of the PWS region. These genetic anomalies were discovered after reexamination of the deletion in later childhood/adolescence. Initial testing in infancy did not revealed this. We did not encounter any case reports similar to our patients 2 and 4, with multiple deletions in the 15q11.2 region. It is striking that in a group of eight patients with atypical deletions, two had this genetic anomaly. Multiple deletions in the region might be more common than initially thought; a first deletion, possibly inherited from a parent, could potentially predispose an individual to a second deletion, contributing to phenotypic variation.

Patient 4 had further peculiarities in her genotype; the MAGEL2 gene was not deleted, but was hypermethylated. In Patient 4, inclusion of the imprinting center in the deletion probably caused the hypermethylation of the MAGEL2 gene, located further upstream. There is one other case in the literature with an atypical deletion encompassing the imprinting center and a hypermethylated pattern of genes upstream and downstream of the deletion [16]. That patient had severe infantile hypotonia and feeding difficulties, skin-picking, hyperphagia and obesity. Her height at examination was in the 10th centile without GH treatment. However, that patient’s phenotype was not evidently different from that of patients with similar atypical deletions that did not involve the imprinting center [11,12,14,15]. This might imply that the severity of the phenotype might not be affected by inclusion of the imprinting center in the deletion, with hypermethylation of the genes outside of the deletion as a result. This is in agreement with our previous conclusion that larger deletions, extending further than SNURF-SNRPN and SNORD116, do not result in a more severe phenotype.

When combining information from our cases and those described in the literature, it seems that in most patients both SNURF-SNRPN and SNORD116 were affected. In more rare cases, only SNURF-SNRPN or SNORD116 were affected, but most of the major features of PWS were found in patients with losses encompassing both genes. Patients with a deletion exclusively in either SNURF-SNRPN or SNORD116 were often atypical and lacked specific symptoms, such as short stature and intellectual disability. Patients with deletions that encompassed only SNURF-SNPRN and SNORD116 did not have a less severe phenotype than patients who had larger deletions in the PWS region. This suggests that both SNURF-SNRPN and SNORD116 contribute to the PWS phenotype, but that genes downstream and upstream of these genes might be less important. Conversely, both genes are produced from the same primary transcript [40], which means that variants in SNURF-SNRPN could affect the expression of SNORD116. No involvement of the imprinting center in the deletion might cause a milder phenotype, whereas inclusion of the imprinting center would have an effect on the expression of other genes in the region; therefore, they might cause a more severe phenotype [16]. However, in the population we studied, patients with a deletion encompassing the imprinting center did not have an evidently more severe phenotype than other patients did.

Differences in phenotypic expression between patients might be caused by factors other than the involvement of specific genes in the deletion. Genes in the region might interact to cause a complex phenotype without one individual gene giving rise to one distinct, separate feature. Furthermore, differences in cognitive functioning between patients may be caused not only by the 15q11.2 deletion, but also by hereditary factors as seen in the general population [41]. Finally, differences in phenotypic expression between patients with PWS might arise from abnormalities in methylation in other chromosomal regions outside of the 15q11.2 locus [42,43].

Growth hormone (GH) treatment influences the natural course of PWS [44,45,46]. As most of our patients were treated with GH, this may have influenced the presence and severity of PWS symptoms. In particular, patients who started GH at a very young age might have a milder phenotype [47]. Patients with an atypical deletion that may be missed by a conventional PWS methylation test could have a delayed diagnosis. These patients would have received less care and treatment for PWS and might, therefore, present with a more severe PWS phenotype. Thus, a severe or mild phenotype might not always be a direct result of a specific genotype, but could be due to differences in received care and treatment.

In summary, we report eight patients with atypical deletions in the PWS region. Our findings further support evidence that even small deletions of single genes or clusters in the region can present with a PWS phenotype, which will often lack several distinctive characteristics. Patients with a deletion limited to SNURF-SNPRN often present with intellectual disability, but do not have short stature. Furthermore, the size of the deletion does not linearly correlate with the severity of symptoms. Even a small deletion can present with a severe PWS phenotype. Comparing our cases to other cases in the literature did not result in a more evident phenotype/genotype correlation; however, it suggests that both SNURF/SNRPN and SNORD116 play an important role in causing the PWS phenotype. This reinforces the concept that genes in the PWS critical region form a complex, interactive network, and that changes in the methylation pattern can cause a diverse phenotype. Small deletions in genes other than SNURF-SNPRN that may be missed by conventional PWS methylation testing can be identified using targeted next generation sequencing. More research is required to narrow down the PWS critical region. An explanation for the enigma of the phenotypic diversity in PWS might be found outside of the 15q11.2 locus and in differentially methylated regions in other parts of the genome.

## Figures and Tables

**Table 1:** Clinical data for patients with an atypical deletion in the PWS region.

	Total	Patient 1	Patient 2	Patient 3	Patient 4	Patient 5	Patient 6	Patient 7	Patient 8
Sex (male / female)	6/2	male	male	male	female	female	male	male	male
Age at diagnosis (weeks)	42.0 (3.4; 103.0)	200	29	55	2.6	94	3.3	3	106
Neonatal characteristics									
Gestational age	40.6 (38.2; 41.6)	42	42	42	40	41	39	33	38
Birth weight (grams)	2645 (2430; 3145)	2800	2430	3260	2430	2765	2525	2195	3266
Birth weight SDS	−2.00 (−2.59; −0.18)	−2.31	−3.19	−1.15	−2.69	−1.91	−2.08	0.28	0.14
Birth length (cm)	48 (46.5; 48.5)	48	unk	49	47	unk	46	unk	48
Birth length SDS	−1.70 (−1.94; −1.09)	−2.11	unk	−1.48	−1.78	unk	−1.70	unk	−0.69
Hypotonia	8 (100)	+	+	+	+	+	+	+	+
Tube feeding	5 (62.5)	+	+	-	+	-	+	+	-
Duration of tube feeding (weeks)	16.0 (6.0; 20.5)	6	17	NA	6	NA	16	24	NA
Cryptorchidism in males	6 (100)	+	+	+	NA	NA	+	+	+
Characteristics at latest examination									
Age (years)	12.0 (5.4; 24.1)	30.3	24.8	22.0	12.1	11.9	9.1	4.2	4.0
Height SDS	−0.81 (−1.87; 0.56)	−1.27	−1.58	−0.35	−1.97	0.82	−0.20	1.91	−2.09
BMI SDS	1.44 (−0.40; 2.87)	−0.91	−0.11	3.72	1.01	2.53	2.98	1.87	−0.50
Age at start GH treatment (years)	2.5 (0.6; 7.9)	14.9	7.9	3.6	0.7	2.5	0.6	0.6	NA
Height SDS before start GH treatment	−2.42 (−3.05; −0.21)	−3.09	−3.36	−2.94	−2.74	0.03	−0.94	0.04	−2.09
Target height SDS	−0.24 (−0.99; 0.51)	0.56	−0.99	−0.85	0.37	0.37	−1.13	1.13	−0.99
(history of) Obesity	4 (50.0)	+	-	+	-	+	+	-	-
(history of) Hyperphagia	4 (50.0)	-	-	+	-	+	+	+	-
Dysmorphisms	6 (75)	+	+	-	+	-	+	+	+
Hypothyroidism	1 (12.5)	-	+	-	-	-	-	-	-
Behavioral problems	3 (37.5)	-	+	-	+	+	-	-	-
Speech problems	2 (25.0)	-	-	-	-	-	-	+	+
Scoliosis	4 (50.0)	+	-	-	+	-	+	+	-
Skin-picking	2 (25.0)	-	-	+	-	+	-	-	-
Developmental status									
IQ	70.5 (65.5; 89.5)	71	65	95	67	73	70	55	95
VIQ	81.5 (64.0; 94.5)	70	62	95	unk	unk	unk	55	93
PIQ	86.0 (69.3; 99.8)	73	68	99	unk	unk	unk	55	100
Intellectual disability	4 (50.0)	-	+	-	+	-	+	+	-
Social-emotional developmental delay	6 (75.0)	+	+	-	+	-	+	+	-

Data are expressed as median (IQR) or n (%); PWS = Prader-Willi Syndrome; SDS = Standard Deviation Score; unk = unknown; NA = not applicable; BMI = body mass index; GH = growth hormone; IQ = intelligence quotient; PIQ = performance IQ; VIQ = verbal IQ.

## Data Availability

The datasets generated during and/or analyzed during the current study are not publicly available but are available from the corresponding author on reasonable request.
